# Extranodal Natural Killer/T-cell Lymphoma, Nasal Type Occurring After Actinomyces Infection: A Case Report

**DOI:** 10.7759/cureus.55594

**Published:** 2024-03-05

**Authors:** Mitsuhiko Katoh, Toshiyuki Mukai, Masakazu Kuriyama, Shunichi Sugasawa, Kento Koda, Gentaro Nagano, Kazuo Yasuhara

**Affiliations:** 1 Department of Otolaryngology - Head and Neck Surgery, Takeda General Hospital, Aizuwakamatsu, JPN; 2 Department of Otolaryngology - Head and Neck Surgery, NTT Medical Center Tokyo, Tokyo, JPN; 3 Department of Otolaryngology - Head and Neck Surgery, Showa General Hospital, Tokyo, JPN; 4 Department of Otolaryngology - Head and Neck Surgery, The University of Tokyo Hospital, Tokyo, JPN

**Keywords:** epstein-barr virus, nasal septum perforation, cd56, actinomyces infection, extranodal natural killer/t-cell lymphoma

## Abstract

The pathogenesis of extranodal natural killer/T-cell lymphoma (ENKTL) remains largely unknown. Herein, we present a case of ENKTL that may have occurred during the treatment of *Actinomyces* infection. A 69-year-old woman was admitted to our hospital with nasal bleeding, and a nasopharyngeal mass was observed. The patient was diagnosed with *Actinomyces* infection on biopsy, and oral antibiotics were administered. The tumor decreased in size; however, swelling of the nasal mucosa and perforation of the nasal septum were observed. A biopsy revealed a recurrence of *Actinomyces* infection, and oral antibiotics were again administered. The mucosal swelling improved temporarily, but the condition gradually deteriorated. The patient was diagnosed with ENKTL based on a third biopsy. Retrospective evaluation of the biopsies showed that there were no CD56-positive cells in the first specimen; however, the number of CD56-positive cells gradually increased in the second and third specimens. We retrospectively observed the occurrence of ENKTL under chronic inflammatory conditions due to *Actinomyces* infection in this case. In addition, this case suggests that the possibility of malignancy must be considered when managing such patients with *Actinomyces* infection.

## Introduction

Extranodal natural killer/T-cell lymphoma (ENKTL) is a disease that may be characterized by swelling and ulceration of the nasal mucosa, as well as perforation of the nasal septum [1]. Although previous literature has established that the Epstein-Barr virus (EBV) plays an essential role in the pathogenesis of ENKTL, the mechanisms underlying EBV infection of natural killer (NK) and T cells remain largely unknown [2]. In addition, under the condition of *Actinomyces* infection, it is difficult to diagnose malignancy because actinomycosis mimics the tumor [3]. In this report, we present a case of pathologically confirmed ENKTL secondary to *Actinomyces* infection in the nasopharynx and nose.

## Case presentation

**Figure 1:**
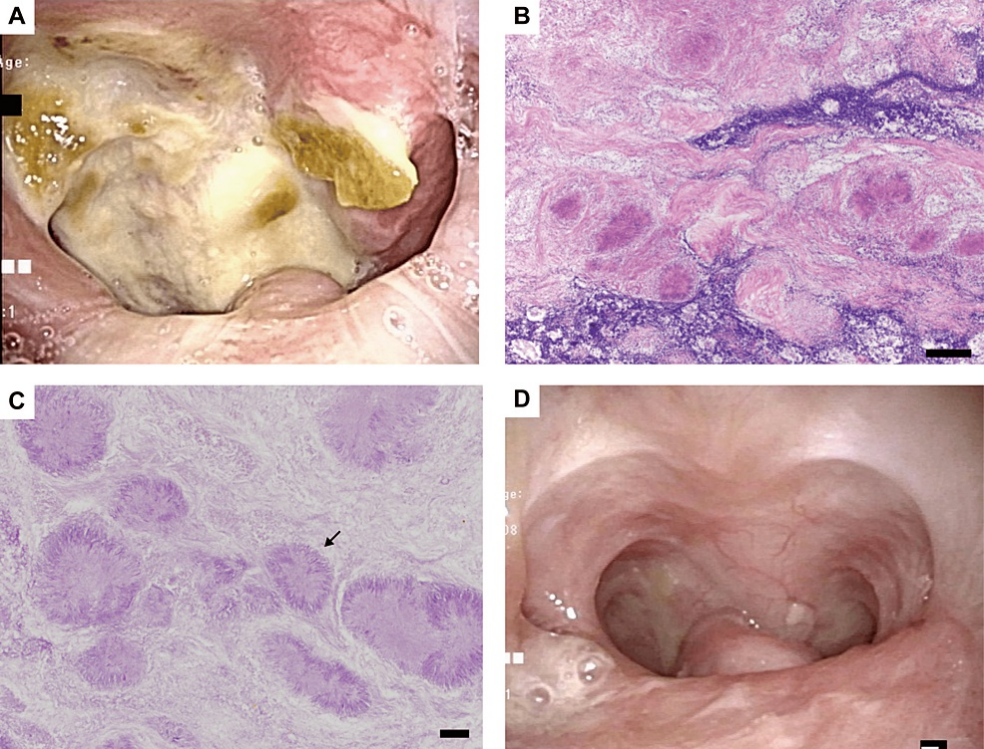
Nasopharyngeal mass with ulceration. (A) Nasopharyngeal ulcer. (B and C) Hematoxylin and eosin staining of the nasopharyngeal ulcer. The bar is 1 mm in (B) and 20 μm in (C). (D) After administration of oral antibiotics, the ulcer resolved. The arrow shows an *Actinomyces* body.

A 69-year-old woman was admitted to our hospital via the ER because of nasal bleeding. She had a history of chronic dacryocystitis. She was not an immunocompromised host and did not take any immunosuppressant. Fiberoptic nasopharyngoscopy (Figure 1A) and computed tomography (CT) scan showed a nasopharyngeal mass with ulceration.

**Figure 2:**
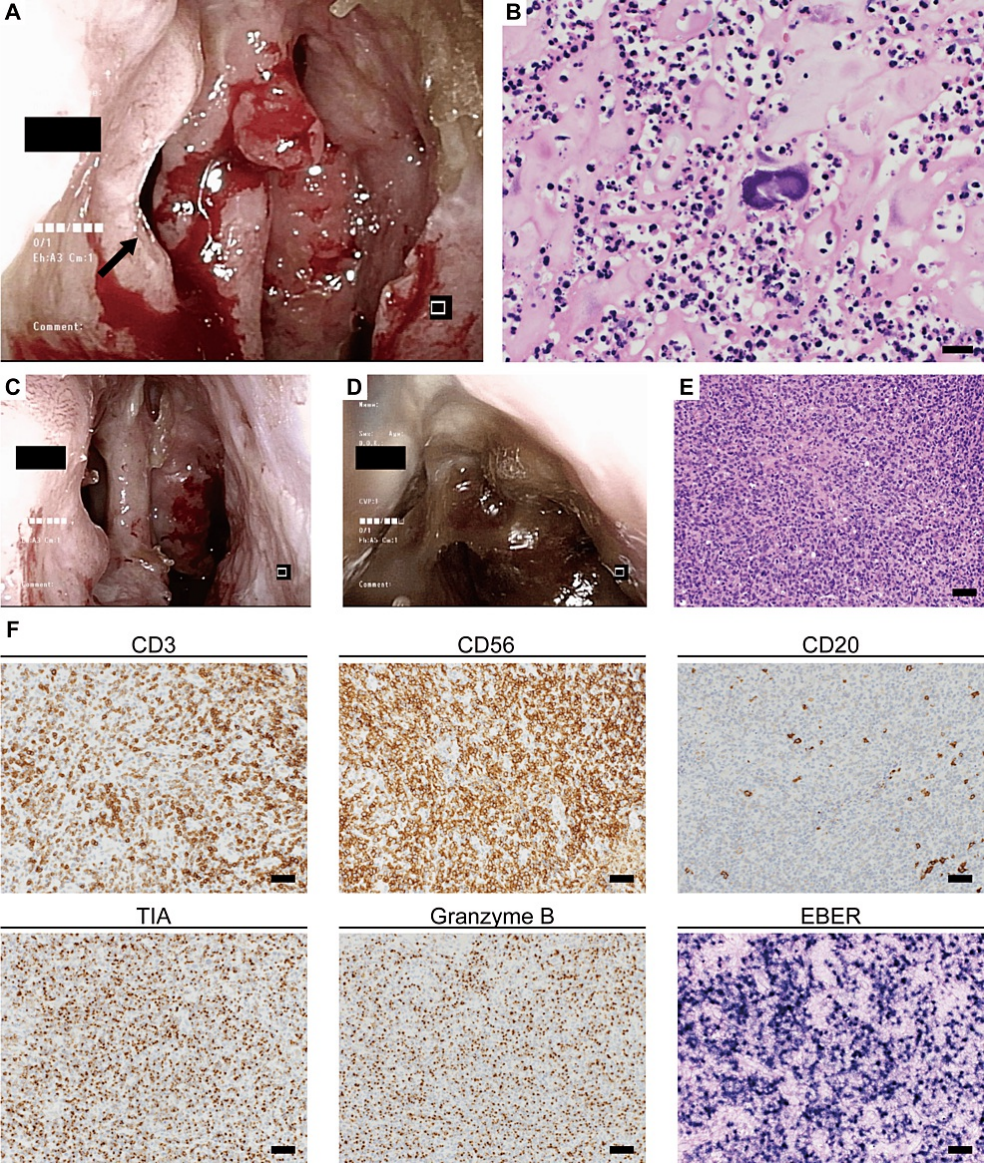
Swelling and ulceration of the nasal mucous membrane and perforation of the nasal septum. (A) Nasal ulceration. The arrow shows perforation of the nasal septum. (B) Hematoxylin and eosin (HE) staining of the nasal ulcer. The bar is 20 μm. (C) After administration of oral antibiotics, the ulcer temporarily improved. (D) Recurrence of the nasal ulcer. (E) HE staining. The bar is 50 μm. (F) Immunostaining and in situ hybridization of Epstein-Barr encoding region (EBER). The bar is 50 μm.

Although sinusitis was observed on the CT scan, the nasal mucosa appeared to be intact. Sphenopalatine artery clipping and a biopsy of the mass were performed under general anesthesia. In the biopsy specimens, several inflammatory cells surrounded *Actinomyces* bodies on staining (Figures 1B, 1C). Thus, we diagnosed the patient with *Actinomyces* infection and initiated oral amoxicillin therapy. Six months after starting amoxicillin, the nasopharyngeal mucosa had almost fully recovered, and antibiotic administration was stopped after six months (Figure 1D). Twelve months after the first admission, swelling and ulceration of the nasal membrane and perforation of the nasal septum were observed on routine follow-up (Figure 2A).

We performed a biopsy again and found many inflammatory cells around the *Actinomyces* bodies in the specimen (Figure 2B). We diagnosed the patient with a recurrent *Actinomyces* infection, and oral amoxicillin was initiated again. It seemed that this recurrence of actinomycosis was not caused by treatment failure or incomplete antibiotics because the infection site was different. Four months after restarting oral antibiotics, the swelling and ulceration of the nasal mucosa had resolved (Figure 2C). Two months following recovery by a four-month antibiotic treatment, the patient experienced nasal pain and swelling, and an ulcer of the nasal mucosa recurred (Figure 2D). Blood tests showed that serum immunoglobulin G4 (IgG4) level was normal, and serum myeloperoxidase antineutrophil cytoplasmic antibody (MPO-ANCA) and proteinase 3 antineutrophil cytoplasmic antibody (PR3-ANCA) assays were negative (Table 1).

**Table 1:** The patient’s blood tests.

Parameters	Value	
C-reactive protein (CRP)	6.33	mg/dL
Immunoglobulin A (IgA)	613	mg/dL
Immunoglobulin G (IgG)	1959	mg/dL
Immunoglobulin G4 (IgG4)	120	mg/dL
Immunoglobulin M (IgM)	39	mg/dL
50% hemolytic complement (CH50)	37	/mL
Complement C3	116	mg/dL
Complement C4	26	mg/dL
Myeloperoxidase antineutrophil cytoplasmic antibody (MPO-ANCA)	Negative	
Proteinase-3 antineutrophil cytoplasmic antibodies (PR3-ANCA)	Negative	
Soluble interleukin-2 receptor (sIL-2R)	657	U/mL

**Figure 3:**
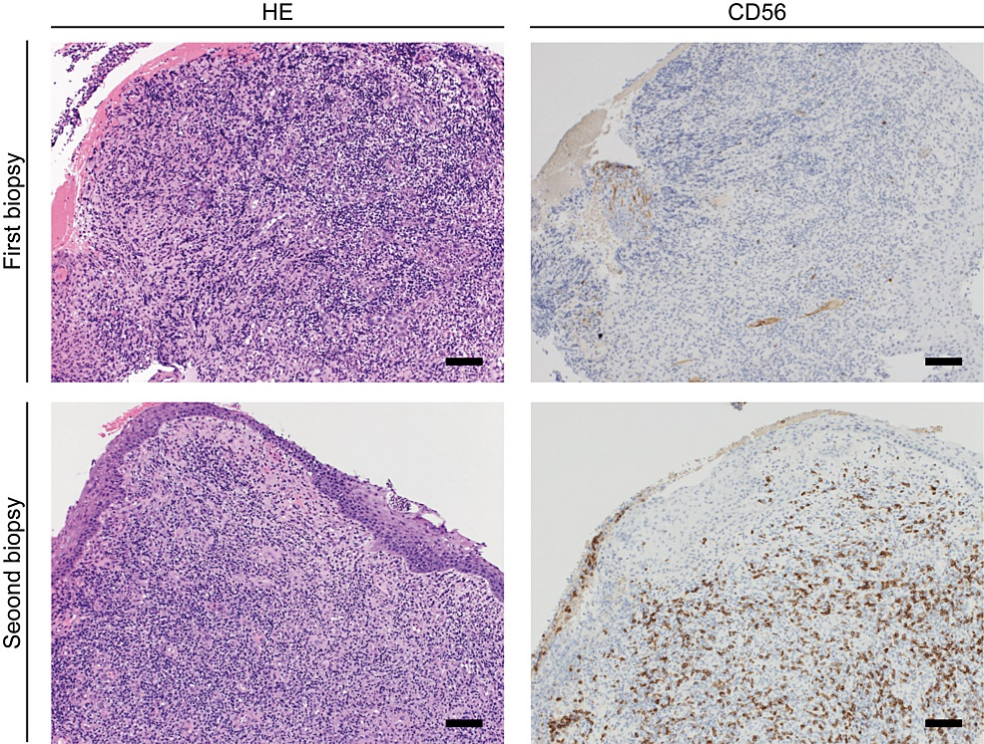
Retrospective review of the first and second biopsies. In the first biopsy, CD56 is negative. In the second biopsy, there are CD56-positive cells. The bar is 100 μm. HE: hematoxylin and eosin.

A biopsy was performed and the specimen was diagnosed as ENKTL (Figures 2E, 2F). The patient was treated with radiotherapy; however, she died of lymphoma in a palliative care hospital five months after diagnosis. We retrospectively reviewed past biopsies; there were CD56-positive cells in the specimen at the first recurrence, whereas no CD56-positive cells were observed in the first specimen (Figure 3).

## Discussion

In the present case report, we described a case of ENKTL that developed after multiple treatments of *Actinomyces* infection. Our retrospective assessment of pathological specimens showed that there were no CD56-positive cells in the first specimen, but CD56-positive cells increased over time in the second and third specimens. These results suggest that ENKTL is affected by chronic inflammation induced by *Actinomyces* infection. Several studies have shown that malignant lymphoma and *Actinomyces* infection can occur simultaneously at the same site. Based on these reports and the present case, the clinical course of *Actinomyces* infection must be carefully observed because it can conceal malignant tumors, especially malignant lymphoma.

In this case, we performed biopsies at every recurrence and retrospectively confirmed the presence of CD56-positive cells in the pathological specimens. There were *Actinomyces* bodies but no CD56-positive cells in the first specimen. Throughout the clinical course, oral intake of amoxicillin resolved the nasopharyngeal mucosal ulcers, suggesting that only *Actinomyces* infection was present. In the second specimen, CD56-positive cells and *Actinomyces* bodies coexisted. Oral intake of amoxicillin partially ameliorated the nasal lesion; however, the mucosal swelling eventually escalated. This suggests that ENKTL and *Actinomyces* occurred at the same site.

Diagnosing ENKTL is difficult, and more than one biopsy is required to reach the final diagnosis in some cases [4]. In addition, although EBV infection is involved in the occurrence of ENKTL, the mechanisms underlying EBV infection of NK and T cells remain largely unknown. Several reports have suggested that chronic inflammation is essential for infection; however, there is little evidence in clinical cases [5,6]. In the present case, we pathologically confirmed the presence of *Actinomyces*, and no CD56-positive cells were observed in the first specimen. There were many inflammatory cells around the *Actinomyces* bodies, and chronic inflammation was prolonged in this case. Retrospectively, the number of CD56-positive cells increased on subsequent biopsies, and we speculate that chronic inflammation may have induced oncogenesis by EBV infection of NK and T cells.

*Actinomyces* are indigenous oral bacteria that are sometimes difficult to diagnose because they can mimic tumors [3]. *Actinomyces* infection and malignant lymphoma have occurred in the same organ in 12 reported cases, including the present case (Table 2).

**Table 2:** Cases of co-occurrence of Actinomyces infection and malignant lymphoma.

Case	Age (years)	Sex	Site	Histology	Report	Year
1	60	Male	Lung	Lymphocytic lymphoma	Winter et al., 1983 [7]	1983
2	Not available	Not available	Neck	Hodgkin lymphoma	Usnarska-Zubkiewicz et al., 1993 [8]	1993
3	45	Male	Lung	Non-Hodgkin lymphoma	Batt et al., 1996 [9]	1996
4	65	Male	Kidney	Mucosa-associated lymphoid tissue (MALT) lymphoma	Garcia et al., 2007 [10]	2007
5	38	Not available	Lung	Hodgkin lymphoma	Weisshaupt et al., 2014 [11]	2014
6	80s	Male	Soft palate	Diffuse large B-cell lymphoma	Hasegawa et al., 2015 [12]	2015
7	62	Male	Stomach	Diffuse large B-cell lymphoma	Carneiro et al., 2016 [13]	2016
8	67	Male	Neck	Diffuse large B-cell lymphoma	Ghanem et al., 2016 [14]	2016
9	66	Male	Stomach	Peripheral T-cell lymphoma	Waki et al., 2017 [15]	2017
10	70s	Male	Root of tongue	Diffuse large B-cell lymphoma	Hasegawa et al., 2018 [16]	2018
11	64	Male	Stomach	Non-Hodgkin lymphoma	Skuhala et al., 2021 [17]	2021
12	69	Female	Nasopharynx and nose	Extranodal natural killer/T-cell lymphoma	This report	2024

Pathological evidence related to EBV was found in two patients [12], including ours. Previously, it was reported that *Actinomyces* infection and methotrexate-associated lymphoproliferative disease (MTX-LPD) can occur in the same organ [18]. MTX-LPD is known to be closely related to EBV, and this report suggests that *Actinomyces* infection facilitates EBV infection through chronic inflammation. Future studies are needed to unveil the mechanisms underlying the oncogenesis of NK and T cells via EBV infection under conditions of chronic inflammation.

In conclusion, our case shows that *Actinomyces* infection and malignant lymphoma can occur in the same organ, and the possibility of malignant lymphoma should be considered in patients with *Actinomyces* infection.

## Conclusions

Herein, we presented a case of ENKTL occurring after actinomycosis. The pathogenesis of ENKTL is believed to be related to chronic inflammation, and our case shows that EBV infection of NK and T cells is related to chronic inflammation caused by *Actinomyces* infection. In the management of actinomycosis, clinicians should be careful when actinomycosis coexists with malignancies.
